# Divergent cardio-ventilatory and locomotor effects of centrally and peripherally administered urotensin II and urotensin II-related peptides in trout

**DOI:** 10.3389/fnins.2015.00142

**Published:** 2015-04-22

**Authors:** Gilmer Vanegas, Jérôme Leprince, Frédéric Lancien, Nagi Mimassi, Hubert Vaudry, Jean-Claude Le Mével

**Affiliations:** ^1^Institut National de la Santé et de la Recherche Médicale UMR1101, Laboratoire de Neurophysiologie, SFR ScInBioS, Université de BrestBrest, France; ^2^Institut National de la Santé et de la Recherche Médicale U982, UA Centre National de la Recherche Scientifique, Différenciation et Communication Neuronale et Neuroendocrine, Université de RouenMont-Saint-Aignan, France

**Keywords:** urotensin II, urotensin II-related peptides, ventilatory variables, heart rate, blood pressure, locomotor activity, brain, trout

## Abstract

The urotensin II (UII) gene family consists of four paralogous genes called UII, UII-related peptide (URP), URP1 and URP2. UII and URP peptides exhibit the same cyclic hexapeptide core sequence (CFWKYC) while the N- and C-terminal regions are variable. UII, URP1, and URP2 mRNAs are differentially expressed within the central nervous system of teleost fishes, suggesting that they may exert distinct functions. Although the cardiovascular, ventilatory and locomotor effects of UII have been described in teleosts, much less is known regarding the physiological actions of URPs. The goal of the present study was to compare the central and peripheral actions of picomolar doses (5–500 pmol) of trout UII, URP1, and URP2 on cardio-ventilatory variables and locomotor activity in the unanesthetized trout. Compared to vehicle, intracerebroventricular injection of UII, URP1 and URP2 evoked a gradual increase in total ventilation (*V*_TOT_) reaching statistical significance for doses of 50 and 500 pmol of UII and URP1 but for only 500 pmol of URP2. In addition, UII, URP1 and URP2 provoked an elevation of dorsal aortic blood pressure (*P*_DA_) accompanied with tachycardia. All peptides caused an increase in locomotor activity (*A*_CT_), at a threshold dose of 5 pmol for UII and URP1, and 50 pmol for URP2. After intra-arterial (IA) injection, and in contrast to their central effects, only the highest dose of UII and URP1 significantly elevated *V*_TOT_ and *A*_CT_. UII produced a dose-dependent hypertensive effect with concomitant bradycardia while URP1 increased *P*_DA_ and heart rate after injection of only the highest dose of peptide. URP2 did not evoke any cardio-ventilatory or locomotor effect after IA injection. Collectively, these findings support the hypothesis that endogenous UII, URP1 and URP2 in the trout brain may act as neurotransmitters and/or neuromodulators acting synergistically or differentially to control the cardio-respiratory and locomotor systems. In the periphery, the only physiological actions of these peptides might be those related to the well-known cardiovascular regulatory actions of UII. It remains to determine whether the observed divergent physiological effects of UII and URPs are due to differential interaction with the UT receptor or binding to distinct UT subtypes.

## Introduction

Urotensin II (UII) is a cyclic neuropeptide that was originally isolated and purified from the caudal neurosecretory system of the teleost fish *Gillichthys mirabilis* (longjaw mudsucker) on the basis of its smooth muscle-stimulating activity (Bern and Lederis, 1969; Pearson et al., 1980). Recently, it has been demonstrated that UII belongs to a family of structurally related peptides that include UII and UII-related peptides (URPs) called URP, URP1, and URP2 (Lihrmann et al., 2013). UII, URP, URP1, and URP2 exhibit the same cyclic hexapeptide core sequence (CFWKYC) while the N- and C-terminal regions are variable (Conlon, 2008; Lihrmann et al., 2013). In the teleost lineage, all four paralog genes are present but only two of them, UII and URP, are found in tetrapods (Quan et al., 2012; Tostivint et al., 2013). In mammals, UII and URP genes are mostly expressed in cholinergic neurons of the brainstem and spinal cord but variable levels of expression occur in most brainstem nuclei (Vaudry et al., 2015) suggesting that the peptides may exert distinct biological functions. UII and URP mRNAs are also differentially expressed in peripheral tissues, including notably the cardiovascular, renal and endocrine systems (Sugo et al., 2003; Dubessy et al., 2008; Vaudry et al., 2015). UII and URP both activate the UT receptor with the same potency (Sugo et al., 2003) but the two peptides may exert differential modulatory effects due to recruitment of different intracellular signaling pathways (Vaudry et al., 2010). The UT receptor is present in several areas of the brain and spinal cord but also in various peripheral organs including the cardiovascular system, endocrine tissues and kidney (Vaudry et al., 2015). UII exerts a large array of biological effects including regulation of various behaviors, motor and neuroendocrine activities, as well as central and peripheral control of blood pressure and heart rate but much less is known about the biological actions of URPs (Vaudry et al., 2010, 2015). In fish, UII, URPs and the UT receptor are also present in the brain and spinal cord. Pioneer studies have demonstrated that UII-like immunoreactivity is primarily found in cerebrospinal fluid (CSF)-contacting neurons located within the ventral ependyma lining the central canal along the entire length of the spinal cord and the medulla oblongata (Yulis and Lederis, 1986, 1988). These CSF-contacting neurons containing UII-like immunorecativity project their axons toward the external surface of the spinal cord and ascending fibers innervate various regions of the brain (Yulis and Lederis, 1986, 1988). UII has been purified and characterized from extracts of the brains of an elasmobranch, the skate *Raja rhina*, and a teleost, the rainbow trout *Oncorhynchus mykiss* (Waugh and Conlon, 1993). The expression of UII mRNA in fish brain has been confirmed by RT-PCR in the European flounder *Platichthys flesus* (Lu et al., 2006), in the zebrafish *Danio rerio* (Parmentier et al., 2008) and in the orange-spotted grouper *Epinephelus coioides* (Sun et al., 2014). Extensive studies on the differential expression of URP, URP1 and URP2 in the central nervous system (CNS) have been conducted in the Japanese eel *Anguilla japonica* (Nobata et al., 2011) and in the zebrafish (Parmentier et al., 2011; Quan et al., 2015). In zebrafish, URP mRNA is present in motoneurons (cited in Quan et al., 2015). In both species, URP1 is mainly expressed in motoneurons of the medulla oblongata. In zebrafish, URP2 mRNA is found in cells located along the ventral edge of the fourth ventricle, probably in CSF-contacting neurons, and in the spinal cord, URP1 and URP2 mRNAs co-localize in same cells that are also CSF-contacting neurons (Quan et al., 2015). In the flounder (Lu et al., 2006), the killifish *Fundulus heteroclitus* (Evans et al., 2011) and the orange-spotted grouper (Sun et al., 2014), the UT receptor is strongly expressed in the caudal neurosecretory system, the CNS (brain and spinal cord) but also in various peripheral tissues including the heart, gill, kidney and ovary. UII is known to be involved in osmoregulation in fish (Marshall and Bern, 1979; Lu et al., 2006; Evans et al., 2011) and a few studies have examined the cardiovascular effects of UII and URP1 in teleosts. In the rainbow trout, centrally administered UII evokes an increase in dorsal aortic blood pressure (*P*_DA_) with variable action on the heart (Le Mével et al., 1996), while intra-arterial (IA) injection of UII provokes a dose-dependent elevation in *P*_DA_ with a concomitant bradycardia (Le Mével et al., 1996). In the Japanese eel, the cardiovascular effects of centrally and peripherally injected UII and URP1 are quite similar. Both peptides preferentially elevate blood pressure in the ventral aorta than in the dorsal aorta and evoke tachycardia (Nobata et al., 2011). In addition, in the rainbow trout, central injection of UII produces a hyperventilatory response and a long-lasting increase in locomotor activity (Lancien et al., 2004). Nonetheless, due to the relatively recent discovery of UPRs, nothing is known about the potential actions of URP1 on ventilatory and locomotor functions and those of URP2 on cardio-ventilatory and locomotor functions. The differential although similar expression of UII, URP1, and URP2 in the CNS of teleosts suggests that these peptides may have synergistic or divergent biological effects. It is thus important to determine the *in vivo* integrative actions of these peptides on physiological functions and behavior in the same animal. Therefore, the main goal of the present study was to analyze the central effects of trout UII, URP1, and URP2 on ventilatory and cardiovascular functions and on locomotor activity in our established trout model. To this end, we have analyzed the effects of intracerebroventricular (ICV) administration of synthetic replicates of these peptides on ventilatory amplitude (*V*_AMP_), ventilatory frequency (*f*_V_), total ventilation (*V*_TOT_), *P*_DA_, heart rate (*f*_H_), and locomotor activity (*A*_CT_). Additionally, the central actions of the peptides were also compared with their effects after IA injection.

## Material and methods

### Peptides and chemicals

The primary sequence of the urotensin peptides examined in this study is shown in Table 1. Trout UII, URP1, and URP2 (Waugh and Conlon, 1993; Tostivint et al., 2013) were synthesized as previously described (Chatenet et al., 2004; Lancien et al., 2004). The peptides were dissolved in Ringer's solution (vehicle) and stored in stock solutions at −25°C. Immediately before use, UII, URP1, or URP2 were diluted to the desired concentration with Ringer's solution. The composition of the Ringer's solution was (in mM): NaCl 124, KCl 3, CaCl_2_ 0.75, MgSO_4_ 1.30, KH_2_PO_4_1.24, NaHCO_3_ 12, glucose 10 (pH 7.8). All solutions were sterilized by filtration through 0.22 μm filters (Millipore, Molsheim, France) before injection.

**Table 1:** **Amino-acid sequence of trout urotensin II (Waugh and Conlon, 1993) and teleost URP1 and URP2 (Tostivint et al., 2013) examined for their cardio-ventilatory effects and locomotor activity following central and peripheral injection in the unanesthetized rainbow trout *Oncorhynchus mykiss***.

**Urotensin II peptides**	**Amino-acid sequence**
UII	H-Gly-Gly-Asn-Ser-Glu-**Cys-Phe-Trp-Lys-Tyr-Cys**-Val-OH
URP1	H-Ala-**Cys-Phe-Trp-Lys-Tyr-Cys**-Val-Thr-Asn-OH
URP2	H-Val-**Cys-Phe-Trp-Lys-Tyr-Cys**-Ser-Gln-Asn-OH

*The conserved cyclic hexapeptide core sequence (Cys-Phe-Trp-Lys-Tyr-Cys or CFWKYC) of each peptide is in bold characters*.

### Animals

Adult rainbow trout *Oncorhynchus mykiss* (247 ± 24 g body wt, mean ± SEM, *n* = 95) of both sexes were purchased locally and transferred in a well-oxygenated and thermostatically controlled water tank to the laboratory. All fish were kept in a 1000-liter tank containing circulating dechlorinated and aerated tap water (11–12°C), under a standard photoperiod (lights on 09:00–20:00). The fish were allowed at least 3 weeks to acclimate under these conditions before the experiments were started. Experimental protocols were approved by the Regional Ethics Committee in Animal Experiments of Brittany, France.

### Experimental procedures

All surgical procedures were made under tricaine methanesulfonate (3-aminobenzoic acid ethyl ester methanesulfonate; 60 mg/L in tap water buffered with NaHCO_3_ to pH 7.3–7.5) anesthesia. The techniques used for placement of the electrocardiographic (ECG) electrodes, placement of the buccal catheter, cannulation of the dorsal aorta and insertion of the ICV microguide have previously been described in detail (Le Mével et al., 1993; Lancien et al., 2004). Briefly, two ECG AgCl electrodes (Comepa, Bagnolet, France) were subcutaneously implanted ventrally and longitudinally at the level of the pectoral fins. The incision was sutured across the electrodes and the leads were sutured to the skin. The dorsal aorta was cannulated with a PE-50 catheter (Clay Adams, Le Pont De Claix, France). A flared cannula (PE-160) was inserted into a hole drilled between the nares such that its flared end was resting against the roof of the mouth. This cannula was used to record any changes in buccal ventilatory pressure. The absence of a neocortex in fish allows the accurate placement of the ICV microguide under stereomicroscopic guidance. A 25-gauge needle fitted with a PE-10 polyethylene catheter was inserted between the two habenular ganglia and descended into the third ventricle until its tip lay between the two preoptic nuclei (Le Mével et al., 2009). An obturator was placed at the end of the PE-10 tubing and the cranial surface was covered with hemostatic tissue followed by light quick-curing resin. After surgery, the animals were force-ventilated with dechlorinated tap water until recovery of opercular movements and transferred to a 6-liter blackened chamber supplied with dechlorinated and aerated tap water (10–11°C) that was both re-circulating and through-flowing. Oxygen partial pressure within the water tank (*P*wO_2_) and pH were continuously recorded and maintained at constant levels (*P*wO_2_ = 20 kPa; pH = 7.4–7.6). A small horizontal aperture was made along the upper edge of the chamber in order to connect the ECG leads to an amplifier and to connect the dorsal aorta and the buccal cannula to pressure transducers. This aperture also permitted ICV and IA injections of peptides without disturbing the animals.

Trout were allowed to recover from surgery and to become accustomed to their new environment for 48–72 h. Each day, the general condition of the animals was assessed by observing their behavior, checking the ventilatory and the cardiovascular variables, and measuring their hematocrit. Animals that did not appear healthy, according to the range of values detailed in our previous studies, were discarded. After stable *V*_AMP_, *f*_V_, *P*_DA_, and *f*_H_ were maintained for at least 90 min, parameters were recorded for 30 min without any manipulation in control experiments. To minimize the use of experimental animals, some trout received both ICV and IA injections. In this later case, the delay between the two injections was 1 day, and the order of the injections was randomized among animals. No single fish was studied for more than 2 days and control experiments revealed that there was no significant change in performance over this period.

### Intracerebroventricular administration of peptides

The injector was introduced within the ICV guide prior to the beginning of a recording session which lasted 30 min. All injections were made at the fifth minute of the test but the injector was left in place for a further 5 min to allow for complete diffusion of the agent and to minimize the spread of substances upwards in the cannula tract. The fish received first an ICV injection of vehicle (0.5 μl) and 30 min later, an ICV injection of UII, URP1, or URP2 (5, 50, and 500 pmol in 0.5 μl). The rationale for using these doses was that they were in the same range as those previously used for studies on the cardiovascular effects of UII in trout and for comparison of effects between peptides (Le Mével et al., 1996, 2012). Previous control experiments using two ICV injections 30 min apart have shown no time-dependent changes in the measured variables using this protocol (Le Mével et al., 2009). The animals received no more than two ICV injections of peptide per day with a delay of at least 5 h between the injections.

### Intra-arterial administration of peptides

Five minutes after the beginning of the recording session, 50 μl of vehicle, UII, URP1, or URP2 at doses of 5, 50, and 500 pmol was injected through the dorsal aorta and immediately flushed by 150 μl of vehicle.

### Data acquisition and analysis of cardio-ventilatory variables and motor activity

The ECG electrodes were connected to a differential amplifier (band pass: 5–50 Hz; Bioelectric amplifier, Gould & Nicolet, Courtaboeuf, France) and a stainless steel bar was immersed in the water of the tank to act as a reference electrode. The aortic cannula and the buccal catheter were connected to P23XL pressure transducers (band pass: 0–15 Hz; Gould & Nicolet). These pressure transducers were calibrated each day using a static water column. At the beginning of the experiments, the zero-buccal pressure level was set electronically. The output signals from the devices were digitalized at 1000 Hz and visualized on the screen of a PC using PowerLab 4/30 data acquisition system (ADInstruments, Oxford, England) and LabChart Pro software (v.7.0; ADInstruments, Oxford, England) during the 30-min recording period and the data were stored on a disk. The time-series related to the ventilatory, the pulsatile *P*_DA_ and the ECG signals were then processed off-line with custom-made programs written in LabView 6.1 (Laboratory Virtual Instrument Engineering Workbench, National Instruments, Austin, USA). Motor activity, ventilatory and cardiovascular variables were calculated as previously described (Lancien et al., 2004; Le Mével et al., 2007). Motor activity was detected as artifacts on the ventilatory signal (Lancien et al., 2004) and the total duration of locomotor activity (*A*_CT_, in seconds) was determined from ventilatory signal (Lancien et al., 2004). Thereafter, segments free of any movement artifacts on the ventilatory signal were selected and *f*_V_ (breaths min^−1^) and *V*_AMP_ (arbitrary units, a.u.) were determined. The *f*_V_ was calculated from the first harmonic of the power spectrum of the ventilatory signal using the fast Fourier transformation. *V*_AMP_ was calculated from the difference between the maximal abduction phase and the maximal adduction phase for each ventilatory cycle. Spontaneous coughings, which correspond to rapid and robust changes in the abduction/adduction phases of the ventilatory cycle, were excluded from this analysis. The net effect of the changes in *f*_V_ and *V*_AMP_ on ventilation was estimated according to the formula *V*_TOT_ = *f*_V_ × V_AMP_, where *V*_TOT_ (a.u.)is total ventilation. Mean *P*_DA_ (kPa) was calculated from the pulsatile *P*_DA_ as the arithmetic mean between systolic blood pressure and diastolic blood pressure, and the *f*_H_ (beats min^−1^) was determined from the ECG signal. All calculations for mean *f*_V_, *V*_AMP_, V_TOT_, *P*_DA_, *f*_H_, and *A*_CT_ were made for the pre-injection period (0–5 min) and for five post-injection periods of 5 min for each trout. To reduce the amount of data, only the maximal effects of the various treatments in the above parameters were analyzed and the results were averaged for trout subjected to the same protocol. One-Way ANOVA analysis of baseline values of *f*_V_, *V*_AMP_, *V*_TOT_, *P*_DA_, *f*_H_, and *A*_CT_ during the pre-injection period revealed that there was no statistical difference between groups prior ICV or IA injection of vehicle or the various peptides (not shown).

### Statistical analysis

Data are expressed as means + SEM (standard error of the mean). The data were analyzed by One-Way ANOVA test followed by the multiple comparison tests of Dunnett or Tukey. The criterion for statistical difference between groups was *P* < 0.05. The statistical tests were performed using GraphPad Prism 5.0 (GraphPad, San Diego, USA).

## Results

### Ventilatory, cardiovascular and locomotor activity responses to central UII, URP1, and URP2

**Figure 1:**
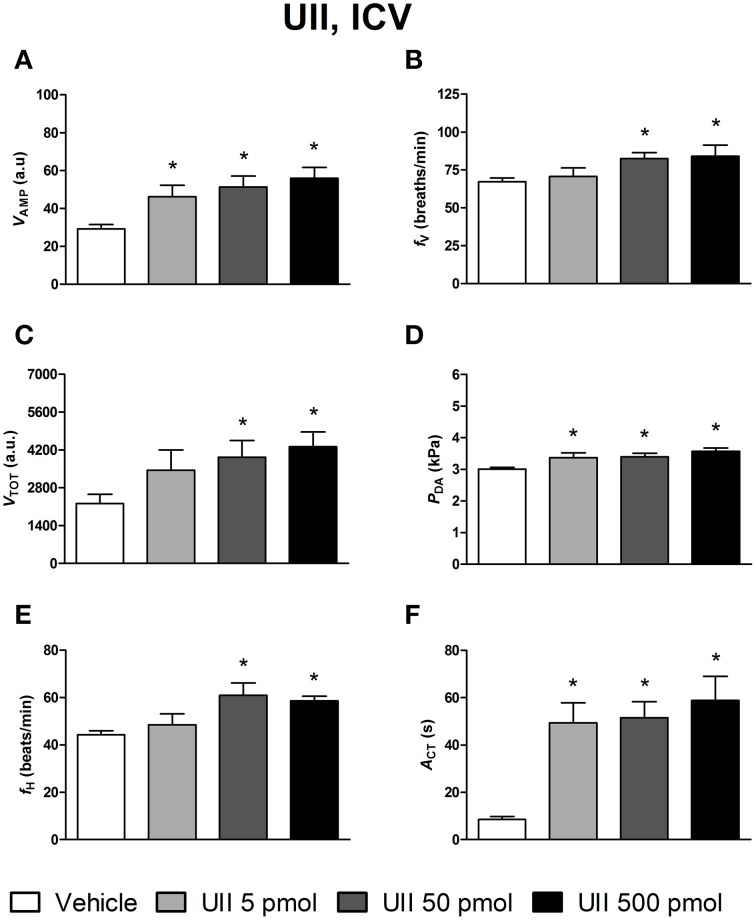
**Histograms showing the maximal effects of ICV injection of 0.5 μl of vehicle (*n* = 34), 5 pmol UII (*n* = 11), 50 pmol UII (*n* = 14) and 500 pmol UII (*n* = 12) on ventilatory amplitude (*V*_AMP_, A), ventilatory frequency (*f*_V_, B), total ventilation (*V*_TOT_, C), dorsal aortic blood pressure (*P*_DA_, D), heart rate (*f*_H_, E), and motor activity (*A*_CT_, F)**. ^*^P < 0.05 vs. vehicle injection.

**Figure 2:**
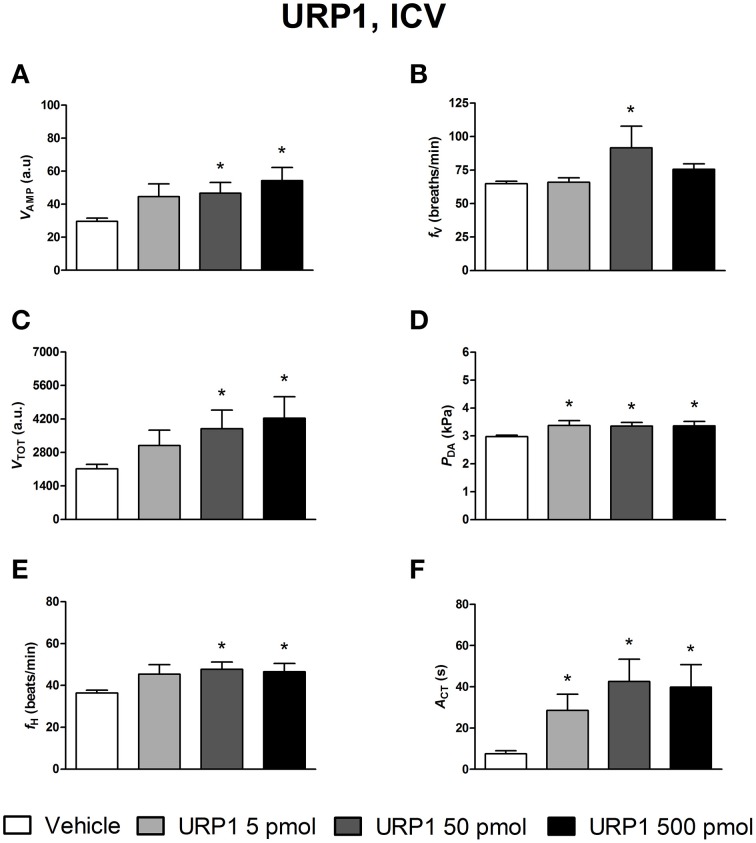
**Histograms showing the maximal effects of ICV injection of 0.5 μl vehicle (*n* = 31), 5 pmol URP1 (*n* = 11), 50 pmol URP1 (*n* = 12) and 500 pmol URP1 (*n* = 10) on ventilatory amplitude (*V*_AMP_, A), ventilatory frequency (*f*_V_, B), total ventilation (*V*_TOT_, C), dorsal aortic blood pressure (*P*_DA_, D), heart rate (*f*_H_, E), and motor activity (*A*_CT_, F)**. ^*^P < 0.05 vs. vehicle injection.

**Figure 3:**
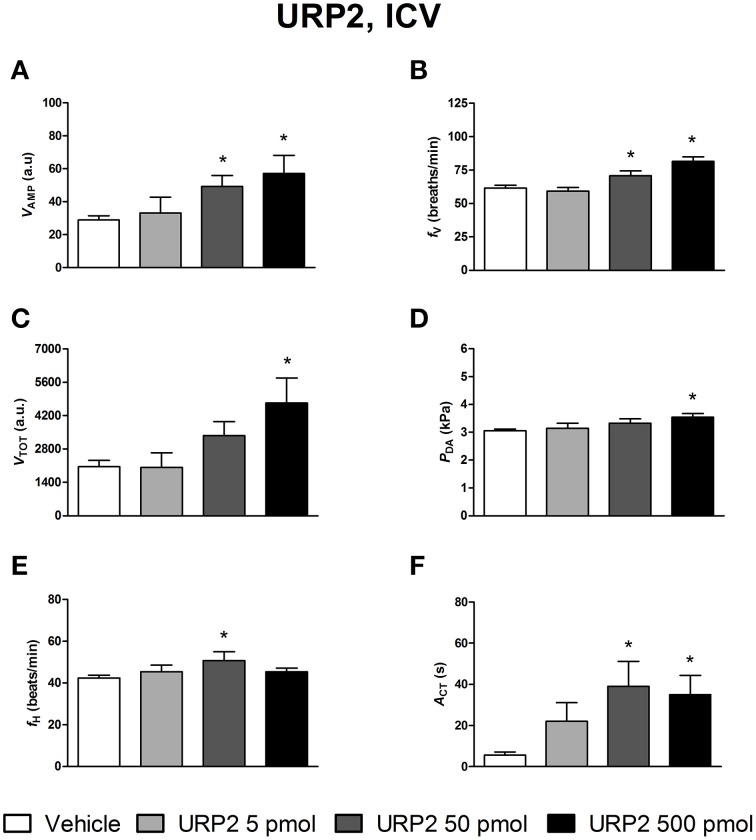
**Histograms showing the maximal effects of ICV injection of 0.5 μl vehicle (*n* = 25), 5 pmol URP2 (*n* = 9), 50 pmol URP2 (*n* = 12) and 500 pmol URP2 (*n* = 9) on ventilatory amplitude (*V*_AMP_, A), ventilatory frequency (*f*_V_, B), total ventilation (*V*_TOT_, C), dorsal aortic blood pressure (*P*_DA_, D), heart rate (*f*_H_, E), and motor activity (*A*_CT_, F)**. ^*^P < 0.05 vs. vehicle injection.

The effects of ICV injections of vehicle, UII, URP1, and URP2 on ventilatory and cardiovascular variables, and on locomotor activity are summarized in Figures 1–3, respectively. Compared with ICV injection of vehicle, all peptides evoked quite similar increase in *V*_AMP_ (Figures 1A, 2A, 3A) and *f*_V_ (Figures 1B,2B,3B). However, the threshold dose of UII inducing a significant effect on *V*_AMP_ was only 5 pmol while a 10-fold higher dose was required for URP1 and URP2. All peptides provoked an elevation of *f*_V_ for a threshold dose of 50 pmol with minor differences between peptides at the higher dose (Figures 1B,2B,3B). The net effect of the ICV administration of the peptides was a hyperventilatory response involving a gradual and significant increase in *V*_TOT_ for doses of 50 and 500 pmol of UII and URP1 but for only 500 pmol of URP2 (Figures 1C,2C,3C). In addition, UII, URP1, and URP2 provoked a non-dose-dependent increase in *P*_DA_ (Figures 1D,2D,3D). However, the threshold dose for this effect was only 5 pmol for UII and URP1 (Figures 1D, 2D) but 500 pmol for URP2 (Figure 3D). During this hypertensive effect of the peptides, there was no bradycardia but instead, a significant tachycardia occurred at the 50 and 500 pmol doses for most of the peptides (Figures 1F,2F,3F). UII, URP1, and URP2 also caused a potent increase in *A*_CT_ for a threshold dose of 5 pmol for UII and URP1 but 50 pmol for URP2.

### Ventilatory, cardiovascular and locomotor activity responses to peripheral UII, URP1, and URP2

**Figure 4:**
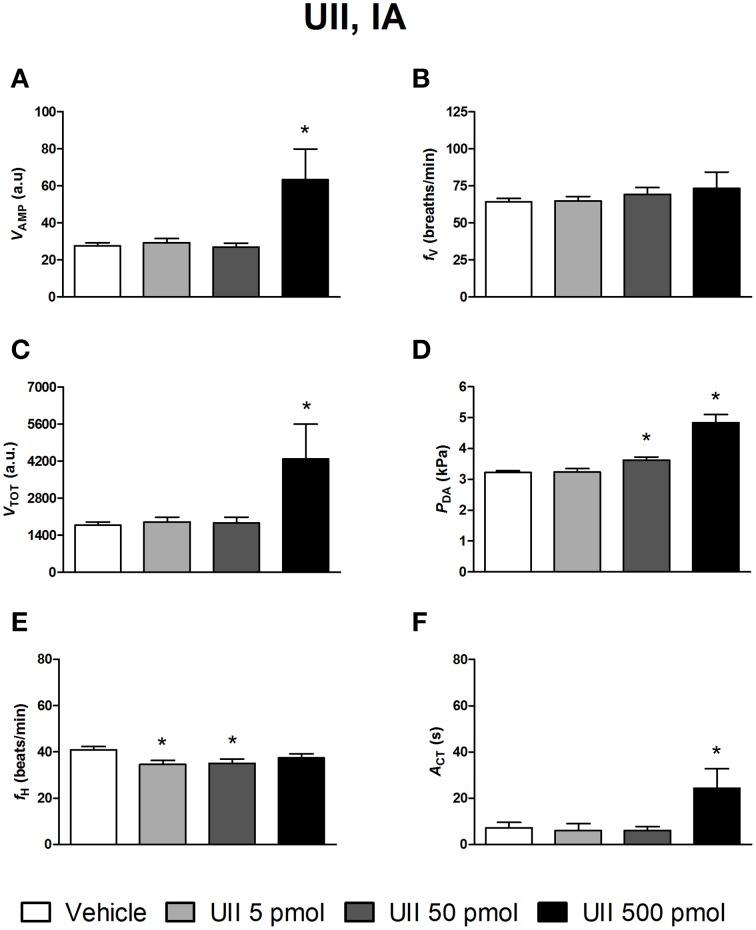
**Histograms showing the maximal effects of IA injection of 50 μl vehicle (*n* = 29), 5 pmol UII (*n* = 10), 50 pmol UII (*n* = 16) and 500 pmol UII (*n* = 7) on ventilatory amplitude (*V*_AMP_, A), ventilatory frequency (*f*_V_, B), total ventilation (*V*_TOT_, C), dorsal aortic blood pressure (*P*_DA_, D), heart rate (*f*_H_, E), and motor activity (*A*_CT_, F)**. ^*^P < 0.05 vs. vehicle injection.

**Figure 5:**
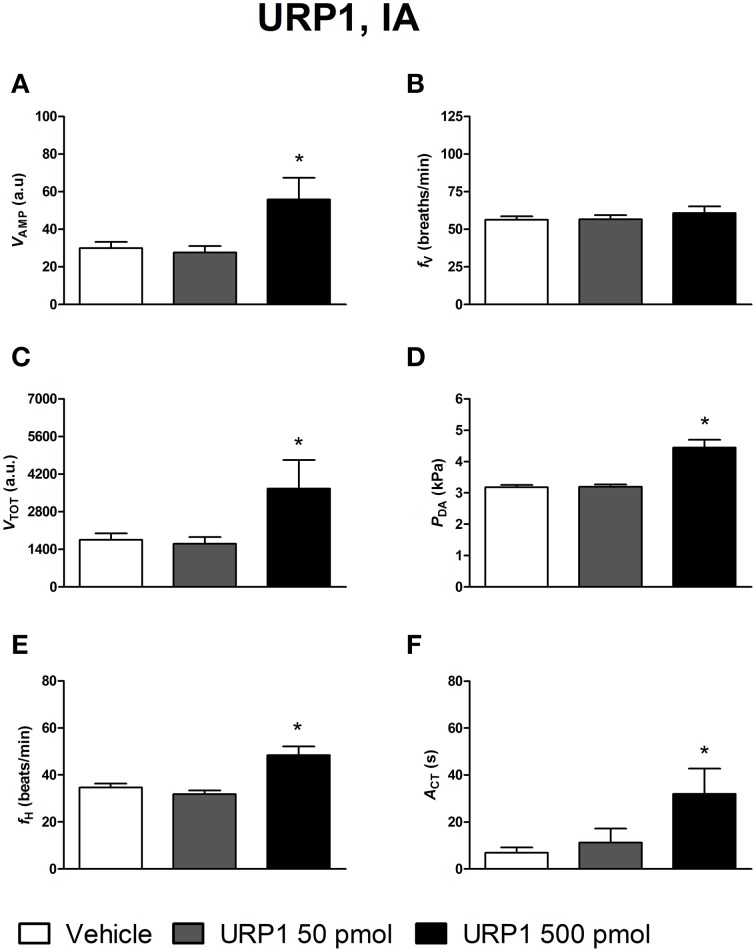
**Histograms showing the maximal effects of IA injection of 50 μl vehicle (*n* = 20), 50 pmol URP1 (*n* = 15) and 500 pmol URP1 (*n* = 11), on ventilatory amplitude (*V*_AMP_, A), ventilatory frequency (*f*_V_, B), total ventilation (*V*_TOT_, C), dorsal aortic blood pressure (*P*_DA_, D), heart rate (*f*_H_, E), and motor activity (*A*_CT_, F)**. ^*^P < 0.05 vs. vehicle injection.

**Figure 6:**
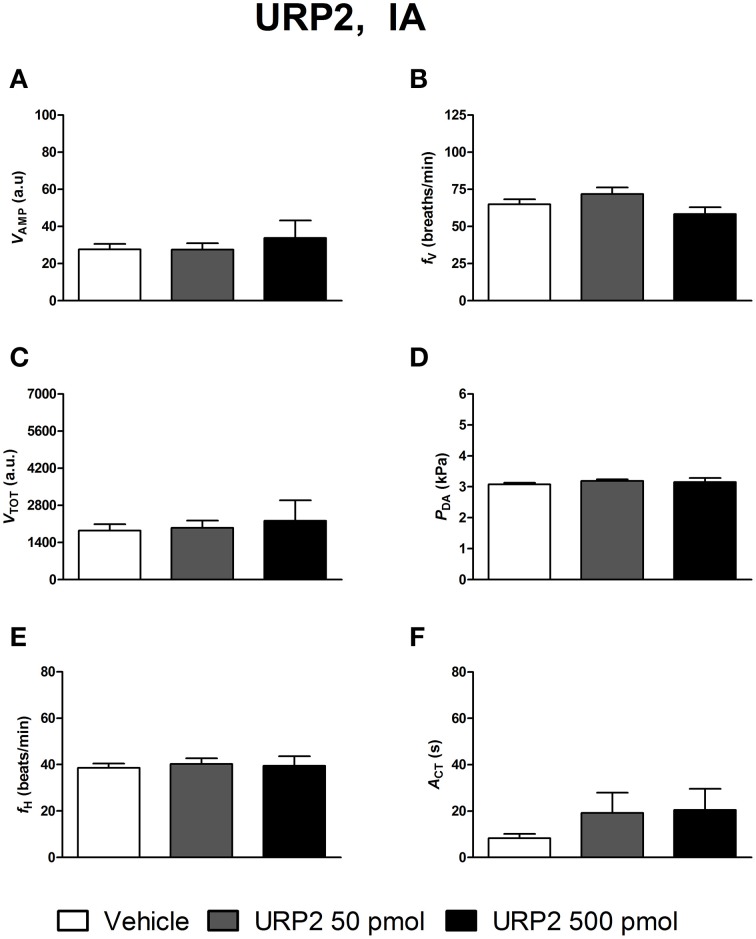
**Histograms showing the maximal effects of IA injection of 50 μl vehicle (*n* = 19), 50 pmol URP2 (*n* = 11) and 500 pmol URP2 (*n* = 9) on ventilatory amplitude (*V*_AMP_, A), ventilatory frequency (*f*_V_, B), total ventilation (*V*_TOT_, C), dorsal aortic blood pressure (*P*_DA_, D), heart rate (*f*_H_, E), and motor activity (*A*_CT_, F)**.

Figures 4–6 depict the results obtained after IA administration of the different peptides on ventilatory and cardiovascular variables and on motor activity. In contrast to their central effects, only the highest dose of UII and URP1 (500 pmol) significantly elevated *V*_AMP_ and the net effect of these peptides was a hyperventilatory response since *V*_TOT_ significantly increased (Figures 4C,5C). Contrary to its ICV effects, IA injection of UII produced a significant dose-dependent increase in *P*_DA_ (Figure 4D) accompanied with a *f*_H_ decrease, a bradycardia statistically significant for the 5 and 50 pmol doses of peptide (Figure 4E). Only the highest dose of URP1 (500 pmol) provoked an elevation in *P*_DA_ accompagnied by a significant tachycardia (Figures 5D,E). IA injection of the highest dose of UII and URP1 caused an increase in *A*_CT_ (Figures 4F,5F). Peripheral administration of URP2 at any dose did not produce any effect on the cardio-ventilatory variables and locomotor activity (Figures 6A–F).

## Discussion

This is the first functional study evaluating the integrative effects of UII, URP1, and URP2 on physiological variables including ventilation, blood pressure and locomotor activity in fish. The most important outcome of this study is that ICV or IA administration of picomolar doses of these peptides exert both common and specific biological activities depending on the route of administration. The demonstration that ICV injection of UII and URPs evoked a stimulatory action on ventilation, cardiovascular variables and locomotion for doses that did not produce any effect or evoked differential action after peripheral administration, supports the assumption that following ICV injection, central neuronal sites are involved in the action of the peptides. In addition, after IA injection, the exclusive effect of low picomolar doses of UII on *P*_DA_ and *f*_H_, confirms that this peptide may act also to peripheral sites.

### Ventilatory, cardiovascular and locomotor actions of centrally administered UII, URP1, and URP2

The central actions of UII, URP1, and URP2 on cardio-ventilatory and motor functions may be compared to those reported in previous studies conducted with UII or URP1 in fish or in other vertebrates species. The present results on UII are consistent with our previous data demonstrating that, in trout, UII administered through the ICV route causes a non-dose-dependent elevation of *P*_DA_ without bradycardia (Le Mével et al., 1996). In the eel, central administration of UII and URP1 preferentially elevates blood pressure of the branchial circulation higher than that of the systemic circulation (Nobata et al., 2011). Consequently, the hypertensive effect of eel UII on ventral aortic blood pressure (*P*_VA_) is significant for doses higher than 0.15 nmol while doses higher than 0.5 nmol are required to increase *P*_DA_. URP1 (0.3–0.5 nmol) provokes a significant dose-dependent increase in *P*_VA_, but does not affect *P*_DA_ at any dose(Nobata et al., 2011). The effect of URP1 was longer lasting than that of UII and the two peptides evoked tachycardia (Nobata et al., 2011). The absence of bradycardia in response to an increase in blood pressure following ICV injection of UII and URP1 in trout and eel, and URP2 in trout suggests that the cardio-inhibitory baroreceptor reflex is altered following central injection of these peptides. In normotensive and hypertensive unanesthetized rats (Lin et al., 2003a,b) and in unanesthetized sheep (Watson and May, 2004), ICV administration of UII causes pressor and tachycardic responses through activation of the sympathetic system indicating that, in these species, also the cardiac baroreflex response is impaired. Studies conducted on unanesthetized sheep to test this hypothesis demonstrated that, after ICV infusion of UII (0.2 nmol/kg for 1 h), the cardiac baroreflex response is effectively blunted since no changes occur in the cardiac sympathetic nerve activity in spite of an increase in blood pressure (Hood et al., 2005). In rats, the central cardiovascular action of UII is site-dependent and local administration of UII in discrete brain nuclei produces differential cardiovascular responses (Lu et al., 2002). To our knowledge, the central action of URP in mammals has never been explored.

We have previously demonstrated that, in addition to its central cardiovascular effects, UII produces a hyperventilatory response and a stimulatory effect on locomotion (Lancien et al., 2004, 2005). In the present study, UII-induced hyperventilation was mimicked by URP1 and to a lesser extent by URP2. Furthermore, at the low dose of 5 pmol, UII, URP1 but not URP2 provoked an increase in locomotion. Nonetheless, at this picomole dose, UII and URP1 did not induced any change in *V*_TOT_. These observations suggest that UII and URP1 act preferentially on central neuronal networks controlling locomotion than ventilation. This stimulatory effect of UII on locomotor activity in fish is in accord with results obtained in rats (Gartlon et al., 2001) and mice (Do-Rego et al., 2005) showing that ICV injection of human UII (hURP, ACFWKYCV) and mouse UII, respectively, elicit motor activity in a familiar environment. It should be noted, however, that the threshold doses eliciting locomotor effects in rats and mice are in the nanomole range. Because UII and URPs induced a marked increased in locomotor activity in trout, we cannot exclude that the changes observed in cardio-ventilatory parameters may be secondary effects of the peptides. Finally, in our study and after ICV injection, a trend in the potency order of UII, URP1, and URP2 emerged being UII ≥ URP1 > URP2 notably for the hyperventilatory, hypertensive and locomotor actions of these peptides.

The receptor site(s) and the multisynaptic pathways involved in initiating cardio-ventilatory and locomotor responses after UII, URP1, and URP2 injection within the brain are matter of speculation and require further studies. Nevertheless, as previously mentioned for the central actions of other neuropeptides, some neuroanatomical prerequisites and some neurophysiological data exist that may support some working hypothesis (Le Mével et al., 2012). Because the peptides were injected within the third ventricle in close proximity to the preoptic nucleus (NPO), they can activate these preoptic neurons leading to hyperventilatory and hypertensive responses through neuroendocrine and/or neurogenic pathways. Preoptic neurons synthesize the nonapeptides vasotocin (AVT) and isotocin (IT). AVT and IT neurons project not only to the neurohypophysis, but also to the brainstem cardiovascular and ventilatory nuclei (Batten et al., 1990; Saito et al., 2004). It should be emphasized that in trout, AVT produces a hypertensive response acting both centrally and peripherally (Le Mével et al., 1993). UII and URPs injected within the third ventricle may also stimulate locomotion through the direct or indirect projection of neurons from the NPO to midbrain locomotor nuclei (Lancien et al., 2004) or spinal motor neurons as previously suggested for the control of sexual behavior (Demski and Sloan, 1985; Gregory and Tweedle, 1985). In addition, since the peptides are injected within the CSF, they can diffuse to the mid- and hindbrain to affect motor nuclei involved in cardio-ventilatory functions and swimming behavior (see also 23). Interestingly, the presence of immunoreactive UII, and URP2 gene expression, in CSF-contacting neurons in regions surrounding notably the fourth ventricular wall, has already been documented in various teleosts (Yulis and Lederis, 1988; Parmentier et al., 2011; Quan et al., 2015), suggesting that these cells may sense the composition of the CSF and/or release their products within the ventricular system. Furthermore, in the eel brainstem, the URP1 gene is detected within neurons of the commissural nucleus of Cajal, a nucleus homologous to the nucleus tractus solitary, the first central relay in the cardiovascular baroreflex loop (Nobata et al., 2011). Concurrently, in the zebrafish, URP1-expressing cells are located in the reticular formation and the glosso-pharyngeal-vagal-motor nuclei (Quan et al., 2015). Collectively, our functional study and these neuroanatomical data support a role of endogenous UII, URP1 and URP2 as neurotransmitters or neuromodulators involved in the central command of autonomic cardio-ventilatory and locomotor functions.

### Ventilatory, cardiovascular and locomotor actions of peripherally administered UII, URP1, and URP2

The peripheral actions of UII, URP1, and URP2 on cardio-ventilatory and locomotor functions may be compared with those reported in previous studies that explored the peripheral effects of UII, URP, or URP1 in fish or in other vertebrate species but also with their central actions. The present results on UII are in line with our previous data obtained in trout demonstrating that low picomole doses of UII cause a dose-dependent hypertensive response and a bradycardia (Le Mével et al., 1996). In addition, we previously investigated the cardiovascular actions of peripherally injected trout UII (50 pmol) and hURP (50 and 500 pmol) in trout (Le Mével et al., 2008). It appears that hURP is about ten times less potent than trout UII in evoking a hypertensive response since hURP (50 pmol, about 0.2 nmol/kg) had no significant effect on cardiovascular variables and only the highest dose of hURP (500 pmol, about 2 nmol/kg) produced a similar peak increase in *P*_DA_. Furthermore, the hypertensive response observed following the IA injection of hURP was of shorter duration than after IA injection of UII and there was no concomitant bradycardia. The cardiovascular effects of UII/URPs in trout are quite different to those obtained in eel, suggesting that the cardiovascular actions of UII/URPs may be species dependent. Indeed, at an equimolar dose of 0.1 nmol/kg in eel, the vasopressor effects of native UII and URP1 are similar, but as for the ICV injection, the effect of UII is longer lasting than the action of URP1. However, in eel, both eel UII and URP1 evoke a tachycardia (Nobata et al., 2011). In addition, after peripheral injections, both hUII and URP are also less potent than the homologous peptides in eel. Thus, the results obtained with heterologous peptides in trout and in eel, emphasize the importance of the amino-acid residues flanking the N-terminus of the cyclic core of the fish UII-molecule in interacting with the fish UT receptor. In trout, the hypertensive effect of UII is mediated through an increase in the systemic vascular resistance since cardiac output decreases (Le Mével et al., 1996). In mammals including humans, a great amount of heterogeneity of vasoactive responses to UII has been observed among vascular beds from species, as well as different regions within the same species (Douglas et al., 2000). The physiological relevance of our experiments may be questioned since the concentration of the injected peptides within the circulation might be more pharmacological than physiological. Consequently, it remains to be determined if physiological plasma concentration of UII may have a role in the cardiovascular regulation in teleosts. In the present study, the highest doses of UII and URP1 but not URP2 evoked cardio-ventilatory and locomotor effects similar to those observed after ICV injection of these peptides. We make the assumption that these effects were mediated through a neurogenic pathway after diffusion of these peptides to critical target sites in the brain that lack the blood-brain barrier (BBB). Some neuroanatomical and functional data favor this hypothesis. At the level of the medulla oblongata, the area postrema is devoid of BBB and acts as a circumventricular organ in the goldfish *Carassius auratus* (Morita and Finger, 1987) and in the eel *Anguilla japonica* (Tsukada et al., 2007). In eel, the organum vasculosum of the lamina terminalis is another circumventricular organ without BBB that may serve as a window for a central action of peripherally injected regulatory peptides (Mukuda et al., 2013). Of interest, this latter organ projects to the NPO. Collectively, these neuro-anatomical data in fish and mammals are consistent with the view that circulating UII, and eventually URP1 but not URP2, may act also as signaling molecules to command some neurally-mediated regulatory mechanisms and notably cardio-ventilatory but also locomotor outputs.

In mammals, the UT receptor is the only high affinity receptor for UII/URP known so far (Vaudry et al., 2015). The UT receptor in teleosts shares about 60% identity with the human UT receptor and, as previously mentioned, is strongly expressed in the caudal neurosecretory system, the CNS and in various peripheral tissues (Lu et al., 2006; Evans et al., 2011; Sun et al., 2014). However, recent data provide evidence for the existence of a vertebrate ancestral UT gene that possessed five distinct UT subtypes in teleosts (Tostivint et al., 2014). The functional role of these receptor subtypes in physiological regulations is currently unknown. It might be questioned whether the divergent physiological effects of UII and URPs after central and peripheral injection observed in the present study may be due to differential interaction with the UT receptor or binding to distinct UT receptor subtypes.

In conclusion, we have examined for the first time in fish the integrative central and peripheral physiological effects of UII, URP1 and URP2 on cardio-ventilatory and locomotor functions. The principal and novel findings of this study are that all peptides produce a central stimulatory effect on ventilation, blood pressure, heart rate and locomotion but with variable potency among peptides. Since the UII, URP1, and URP2 genes are expressed in the CNS, our results suggest that the endogenous peptides may be implicated as neurotransmitters or neuromodulators in the regulation of cardio-ventilatory and locomotor functions in trout. After systemic administration of low picomole doses, none of the UII and URP peptides affect ventilation or locomotion, but only UII evokes hypertension and bradycardia, indicating that endogenous UII may have a role as circulating hormone involved in cardiovascular regulation in trout. Further studies are clearly required to determine under which circumstances the different neuroendocrine and neuronal pathways that mediate the integrative effects of the urotensinergic system are recruited to participate in cardio-ventilatory and locomotor regulations.

### Conflict of interest statement

The authors declare that the research was conducted in the absence of any commercial or financial relationships that could be construed as a potential conflict of interest.

## Abbreviations

*A*_CT_: locomotor activity
a.u.: arbitrary unit
CNS: central nervous system
ECG: electrocardiographic
*f*_H_: heart rate
*f*_V_: ventilatory rate
IA: intra-arterial
ICV: intracerebroventricular
*P*_DA_: dorsal aortic blood pressure
UII: urotensin II
URP: urotensin II-related peptide
URP1: urotensin II-related peptide 1
URP2: urotensin II-related peptide 2
*V*_AMP_: ventilatory amplitude
*V*_TOT_: total ventilation.
